# Different temporal dynamics after conflicts and errors in children and adults

**DOI:** 10.1371/journal.pone.0238221

**Published:** 2020-08-31

**Authors:** Mirela Dubravac, Claudia M. Roebers, Beat Meier

**Affiliations:** Institute of Psychology, University of Bern, Bern, Switzerland; University of Wuerzburg, GERMANY

## Abstract

After perceiving cognitive conflicts or errors, children as well as adults adjust their performance in terms of reaction time slowing on subsequent actions, resulting in the so called post-conflict slowing and post-error slowing, respectively. The development of these phenomena has been studied separately and with different methods yielding inconsistent findings. We aimed to assess the temporal dynamics of these two slowing phenomena within a single behavioral task. To do so, 9-13-year-old children and young adults performed a Simon task in which every fifth trial was incongruent and thus induced cognitive conflict and, frequently, also errors. We compared the reaction times on four trials following a conflict or an error. Both age groups slowed down after conflicts and did so even more strongly after errors. Disproportionally high reaction times on the first post-error trial were followed by a steady flattening of the slowing. Generally, children slowed down more than adults. In addition to highlighting the phenomenal and developmental robustness of post-conflict and post-error slowing these findings strongly suggest increasingly efficient performance adjustment through fine-tuning of cognitive control in the course of development.

## Introduction

Experiences shape our behavior and behavior shapes our experiences. If we experience a difficult task, we generally slow down performance to avoid errors. In laboratory studies, difficulty is induced by cognitive conflicts where an automatic prepotent response has to be inhibited to avoid errors. When experiencing conflicts or errors, adults as well as children slow down performance [1–10]. The ability to adjust our behavior flexibly is critical for achieving our goals and is thought to be a driving force for cognitive development in adolescence [11,12]. Understanding the time course of adjustment processes after experiencing conflicts and errors may elucidate fine-grained cognitive development throughout adolescence. Thus, we investigated the temporal dynamics of performance adjustments after experiencing increased task difficulty induced by occasional cognitive conflicts and errors in preadolescent children and young adults.

One often used experimental task to induce cognitive conflict is the Simon task [6,13,14]. In a variant of this task, colored stimuli appear on the left or right hand side of a screen. Participants are asked to respond to the color of the stimuli (task-relevant dimension) by pressing the corresponding left or right key as fast and accurately as possible while ignoring the location of the stimulus (task-irrelevant dimension). On congruent trials, the task-irrelevant location and the task-relevant color point to the same response. On incongruent trials, however, the task-irrelevant location and the task-relevant color point to different responses. Incongruent trials, thus, induce a response conflict. The costs of this cognitive conflict is reflected in the congruency effect which is characterized by slower reaction times and higher error rates on incongruent compared to congruent trials [15]. Incongruent trials have also longer-lasting effects on performance as evidenced by the **post-conflict slowing** effect [1,2,5]. Similarly, errors also lead to subsequent performance adjustments as evidenced by the **post-error slowing** effect [16].

One prominent framework to account for performance adjustments is the *conflict monitoring theory* [17]. According to this theory, incongruent trials simultaneously activate two conflicting response alternatives leading to response conflict. Detection of response conflict increases cognitive control on the next trial, which leads to increased focusing on the task-relevant stimulus dimension. This increased task-focusing then leads to reduced interference from the task-irrelevant stimulus dimension on the subsequent incongruent trial [6,7,18–21]. It also leads to increased reaction times on several trials after experiencing the conflict, that is, post-conflict slowing [1]. According to the conflict monitoring theory, the detection of a committed error is associated with high levels of conflict as for a short moment both the correct and incorrect responses are co-activated leading to cognitive conflict and subsequent slowing [17,22].

The generalizability of the conflict monitoring theory has been challenged by studies suggesting a dissociation between post-conflict and post-error control adjustments. For example, post-error slowing generalized over task-sets while post-conflict adaptation was task-specific [4,23]. Moreover, different event-related potentials were found for post-error and post-conflict slowing [24]. However, more recent studies suggest that the task-specificity of post-conflict slowing changes across the course of a task [1], and that task-unspecific post-error slowing dissipates quickly while task-specific post-error slowing persists [25,26]. Moreover, evidence for the involvement of two processes with different developmental trajectories is found for post-conflict slowing, as well as post-error slowing [8,27–29]. However, so far, no study has compared the time-courses and developmental trajectories of the two slowing phenomena directly. The aim of the present study was to fill this gap by examining the reaction times on four trials after conflicts and errors in children and adults. Before coming to our own study, we briefly review studies that examined the time-course of post-conflict slowing or post-error slowing in children and/or in adults.

Post-conflict slowing has been investigated by inducing occasional conflict trials (i.e., incongruent trial in the Simon task) and measuring reaction times on subsequent non-conflict trials. The increase in reaction times relative to a block involving only non-conflict trials represents post-conflict slowing [30]. Presenting adult participants incongruent trials on every fifth trial, post-conflict slowing was found on all four subsequent trials [1]. A related study showed even longer lasting post-conflict slowing by presenting incongruent stimuli on six trials evenly interspersed among 120 trials resulting in 19 post-conflict trials [2]. In this study, adults showed post-conflict slowing for up to twelve trials [2]. This method has also been used to investigate post-conflict slowing in children. Monolingual children showed post-conflict slowing for up to twelve trials while bilingual children showed post-conflict slowing only for up to two trials, suggesting faster disengagement of attention by bilingual children [5]. Assuming that adults are faster in regulating attention control compared to children, we hypothesized that children would show stronger and longer lasting post-conflict slowing.

Post-error slowing has been measured by comparing reaction times on post-error trials to either correct trials [9,10,25,31,32], post-correct trials [33–37] or pre-error trials [26,38]. A recent study compared the different measures [39]. Most important for the present study, post-error slowing was found consistently in children as well as in adults. For example, in one study reaction times were examined on ten trials before and after an error in several age groups from five-year-olds to young adults [9]. Participants responded increasingly faster before an error with the fastest reaction times observed on the error trials, after which they slowed down markedly. Reaction time differences between trials decreased with age reflecting a trend towards smoother and more fine-tuned reaction time adjustments across development. A more recent study examined reaction times around errors in the Simon task and corroborated the finding of faster reaction times on pre-error and error trials and stronger post-error slowing in younger compared to older children [40]. In line, other studies also found a decrease in post-error slowing with age [10,31,32,41]. Based on these findings, we assumed stronger and longer lasting post-error slowing in children than adults.

In the present study, children and adult participants completed a Simon task in which every fifth trial was incongruent. We were especially interested in comparing the time courses of post-conflict slowing and post-error slowing. Due to the experimental set up, with every fifth trial being an incongruent trial, errors were provoked experimentally. This method allows for a differentiated investigation of performance slowing for up to four subsequent trials [42]. Post-conflict slowing was measured by subtracting individual reaction times in a pure congruent block from reaction times on the four trials immediately following a *correct* response to an incongruent trial. Aiming to compare the time-courses, post-error slowing was measured correspondingly; reaction times in a pure congruent block were subtracted from reaction times on the four trials immediately following an *erroneous* response to an incongruent trial. Thus, the main analysis focused on congruent trials following correct and incorrect incongruent trials, respectively.

We expected that error specific slowing would add up to conflict related slowing. The difference represents slowing uniquely attributable to the error. Thus, we hypothesized stronger post-error slowing compared to post-conflict slowing. We also hypothesized that the time courses of post-conflict and post-error slowing would be comparable across age groups. Although young children already have the ability to inhibit responses and thus modulate cognitive control [6] further development is taking place during late childhood as older children show more fine-tuned performance adjustments [9,31]. During the maturation of the prefrontal cortex supported by synaptic pruning and myelination adolescents further develop their inhibitory skills and fine-tune performance adjustment skills [12,43–46]. As inhibition and resistance to interference as well as deliberate strategy use develop substantially through childhood and until adulthood, and based on previous findings reported above, we expected stronger slowing in children than adults.

## Method

### Participants

We recruited 65 children from schools of the German speaking part of Switzerland and 75 adults via advertisements on the university’s billboard or posted in a local online news portal. Based on our piloting studies, we assumed more exclusions in the adult sample and thus we recruited more adults than children. After data screening, we excluded twelve adult participants because they did not commit any error on the critical incongruent trials intended to elicit errors. We excluded two children because they responded incorrectly on the critical post-error trials as well. The final sample consisted of 63 children aged between 9 and 12 years (*M* = 11, *SD* = 1, 32 males) and 63 adults aged between 18 and 33 years (*M* = 23, *SD* = 3, 13 males).

The study was approved by the ethics committee of the University of Bern. Prior to testing the participants, we obtained written informed consent of the adult participants and children’s parents. Children received a small present for their participation, psychology students received credits and external participants were financially compensated.

### Design

The study design consisted of the between-subject factor *age group* (child vs. adult) and three within-subject factors; *congruency* (congruent vs. incongruent trials), *slowing type* (post-conflict vs. post-error), and position of the current *trial* (t+1, t+2, t+3, t+4, with t referring to an incongruent trial). Accuracy and reaction times were measured on every trial and provided the basis for computing post-conflict and post-error slowing.

### Materials

**Fig 1:**
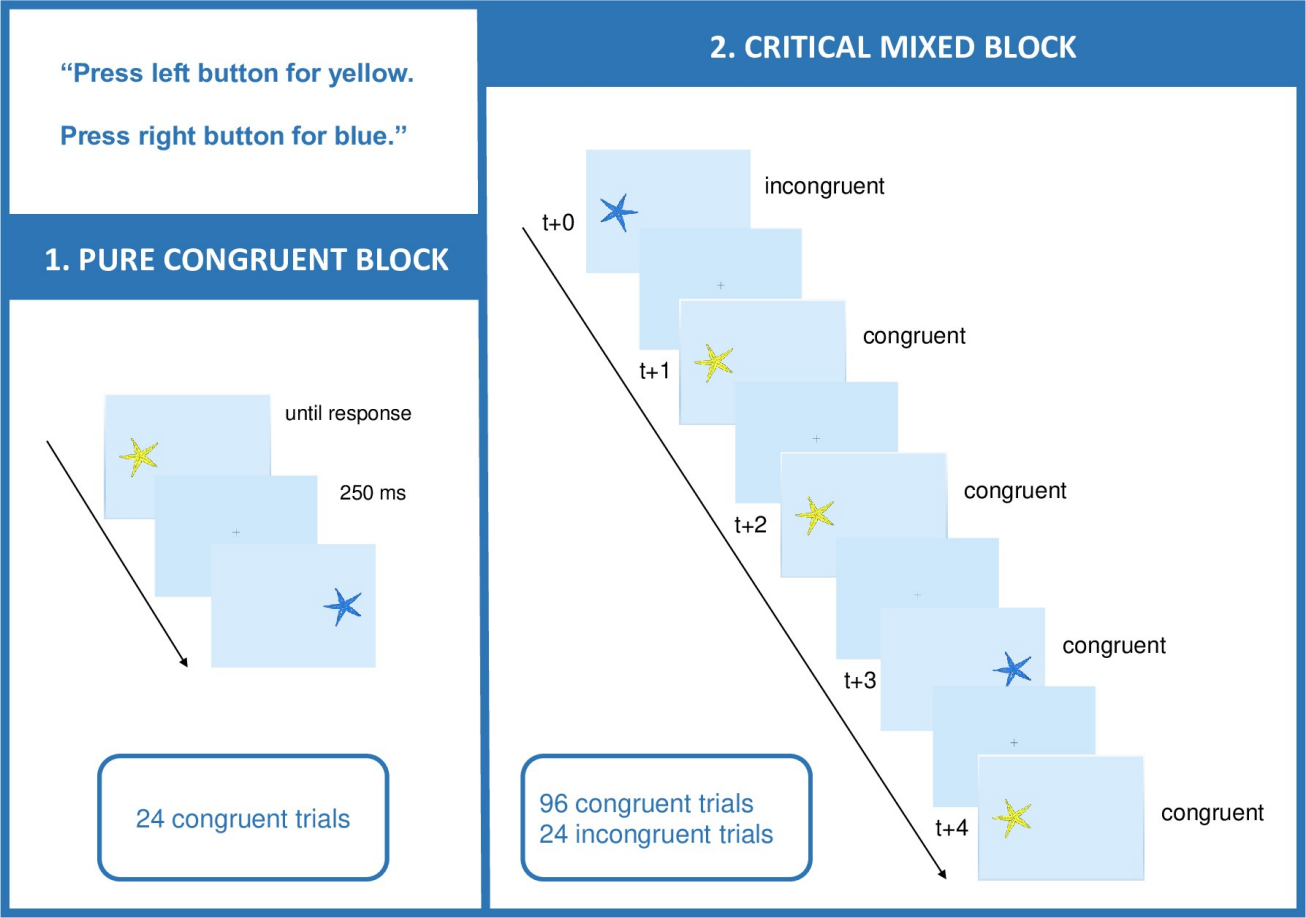
Schematic representation of the procedure with congruent and incongruent trial examples. Procedure; Depending on whether the response on trial t+0 was correct or incorrect, the subsequent trials (t+1, t+2, t+3, t+4) were considered as post-conflict or post-error trials, respectively.

The Simon task was adopted from Roebers and Kauer (2009) [13]. The stimuli were yellow and blue starfish, which appeared on the left or right side of a laptop screen (Fig 1). The stimuli were presented in two blocks with a fixed block order, which was not repeated. The first, purely congruent block comprised 24 trials, in which 12 yellow and 12 blue starfish appeared in random order always on the congruent response side. The second, mixed block comprised a total 124 trials, of which the first four trials were congruent warm-up trials. The remaining 120 experimental trials in the mixed block comprised 96 congruent trials and 24 incongruent trials. On incongruent trials, the starfish appeared on the incongruent response side. The incongruent trials were determined randomly with replacement and were evenly interspersed among the 96 congruent trials, occurring on every fifth trial [42].

### Procedure

The procedure was the same for children and adults. Participants were tested individually. They were seated in front of a laptop computer at approximately arm length distance to the keyboard, on which the response had to be given by pressing either the left mouse button with the left index finger for a yellow starfish or the right mouse button with the right index finger for a blue starfish. Each trial consisted of a fixation cross for 250 ms (= response-stimulus interval) in the middle of the screen, followed by a yellow or blue starfish which appeared either on the left or right side and stayed on screen until response. Participants were asked to respond as accurately and fast as possible. The procedure is depicted in Fig 1.

To ensure that the task was clear to the participants the examiner showed two congruent example trials before the congruent practice block and two incongruent example trials before the mixed practice block. The practice blocks consisted of four practice trials. In the case of more than two errors, the experiment automatically returned to the start of the practice block and the examiner explained the task again (this happened in three cases altogether). After a successful practice run, the respective experimental block started. The mixed block was preceded by four congruent warm up trials not included in the analysis.

The reasons for presenting a pure congruent block before the mixed block were a) to induce a congruency training (i.e., to establish a response tendency to the side of the stimulus) aimed at increasing error rates on incongruent trials in the following mixed block, b) to be able to compute post-conflict slowing by comparing the reaction times on congruent trials between blocks, and c) to have a baseline for speed-differences between children and adults [2]. The pure congruent block consisted of 24 congruent trials, while the mixed block consisted of 24 incongruent trials evenly interspersed among 96 congruent trials. We were mainly interested in the four congruent trials following incongruent trials. Collapsing these four trials to one event results in 24 incidents of performance adjustments after conflicts and errors (120/5 = 24). This in turn can be related to the 24 incidents of congruent trials in the shorter congruent block. With this trial number and block order we successfully created a short, convenient, and child-friendly task inducing a maximal error rate on incongruent trials. While piloting the task, we aimed at a 20% error rate. We reached this goal with the presented method, although the error rate was slightly lower in the present sample (i.e., 17.15%).

### Data preparation and analysis

For every participant, mean accuracy rates and median reaction times in ms were computed. Compared to the mean, the median is less susceptible to outliers. We preferred to keep as many trials as possible in the analysis and thus we used the median. However, conclusions do not change after conducting the analyses with the mean. Because we were interested in the reaction times on *correctly* answered congruent trials after incorrectly answered incongruent trials, we had to exclude participants who did not provide values in those cells. That is, we excluded participants either who did not commit any errors on incongruent trials or who committed further errors on the congruent trials after an error on an incongruent trial.

The pure congruent block served as a baseline for the mixed block which comprised the critical incongruent (conflict) and congruent (non-conflict) trials. As a manipulation check, we analyzed the congruency effect to confirm that the incongruent trials indeed induced cognitive conflict in both age groups. The main analysis applied to reaction times on four congruent trials as a function of the correctness of the response on the previous incongruent trial. We excluded incorrect responses on congruent trials and discarded the following trials as well (thus excluding double errors). This yielded a total of 1907 post-error trials and 9511 post-conflict trials, that is, on average, 69 post-conflict and 19 post-error observations per child, and 82 post-conflict and 11 post-error observations per adult.

In order to control for speed differences, we computed post-conflict and post-error slowing by subtracting individual median correct reaction times in the pure congruent block from the four trials following incongruent trials in the mixed block. A significant difference from zero thus represents conflict and error related slowing (as it is compared to a block without conflict and with very little errors). This is a standard analytical approach used to investigate post-conflict slowing[1,2,5,30]. For comparability reasons, we computed post-error slowing the same way. To compare our findings to other studies of post-error slowing, however, we additionally calculated post-error slowing as the difference between post-error and post-correct reaction times. This measure represents pure post-error slowing because conflict related slowing is subtracted.

We performed analyses of variance (ANOVA) on accuracy rates, reaction times and difference scores representing slowing. For significance tests, an alpha level of .05 was set. As Mauchly’s test of sphericity was significant for the factor trial, corrected Greenhouse-Geisser values are reported.

## Results

### Descriptive statistics

**Fig 2:**
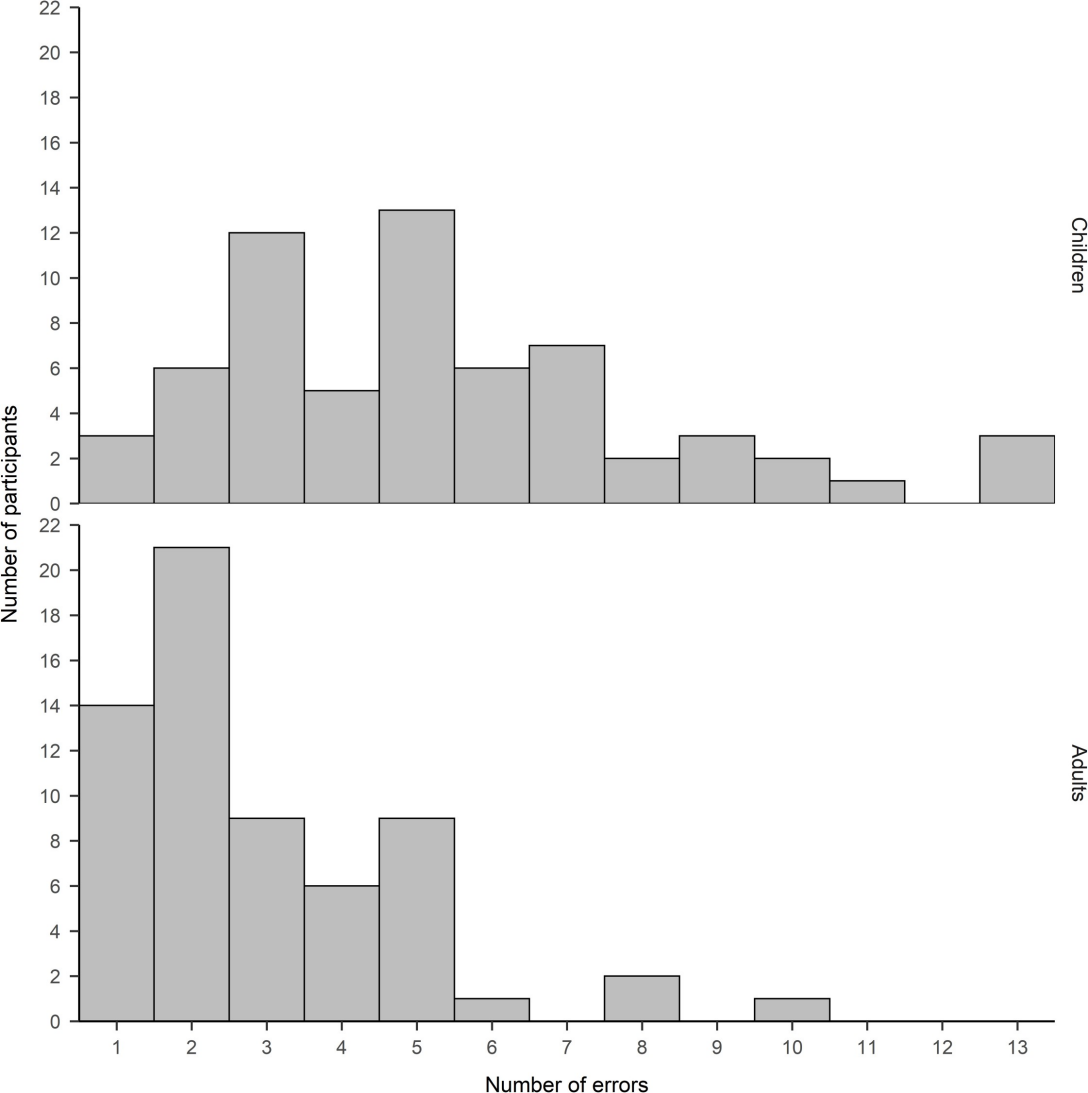
Distribution of error rates on incongruent trials among participants.

Tables 1 and 2 give a descriptive overview of the data. Participants performed well on the congruent trials of the pure block with accuracy rates at ceiling. Critical for assessing post-error slowing in the mixed block, accuracy was below 85% on incongruent trials while accuracy was relatively high on congruent trials. Fig 2 shows the distribution of errors on incongruent trials among children and adults. Not surprisingly, children made more errors than adults. The even distribution of errors among participants suggests that the error rate is not driven by outliers.

**Table 1:** Reaction times and accuracy for pure and mixed blocks.

		Pure block	Mixed block	
Measure	Age group	Congruent	Congruent	Incongruent
RT	Children	388 (9)	598 (10)	733 (15)
	Adults	315 (7)	432 (9)	519 (11)
Accuracy	Children	0.987 (0.003)	0.970 (0.005)	0.779 (0.015)
	Adults	0.999 (0.001)	0.994 (0.001)	0.878 (0.010)

RT = mean of the correct median reaction times in ms, accuracy = mean proportion of correct responses. Standard errors of the means are in parentheses.

**Table 2:** Reaction times in the mixed block.

Age group	Trial type (T)	T+0	T+1	T+2	T+3	T+4
Children	Correct conflict	735 (16)	624 (13)	577 (11)	540 (9)	536 (10)
	Incorrect conflict	485 (13)	874 (32)	645 (21)	560 (12)	591 (15)
	Pure post-error slowing	-250 (14)	249 (29)	68 (18)	20 (10)	56 (13)
Adults	Correct conflict	518 (11)	450 (10)	429 (10)	403 (9)	406 (9)
	Incorrect conflict	355 (13)	667 (40)	482 (19)	436 (13)	446 (15)
	Pure post-error slowing	-163 (11)	218 (33)	52 (16)	33 (10)	40 (11)

Values represent the mean of median reaction times in ms. Standard errors are in parentheses. “T+0” is the incongruent trial and “T+1”, “T+2”, “T+3”, “T+4” denote the following four congruent trials. Please note that the values are not exactly the same as in Table 1 (cf. “incongruent trial in the mixed block” in Table 1 vs. “correct conflict on T+0” in Table 2), due to the exclusion of correct incongruent trials that were preceded by an error. The difference between “correct conflict” and “incorrect conflict” represents “pure post-error slowing” (shaded).

### Congruency effect

To test for the expected congruency effect (longer reaction times and lower accuracy rates on incongruent trials compared to congruent trials), we conducted a 2x2 ANOVA on accuracy rates and correct reaction times from the mixed block with the between-subjects variable age group (children vs. adults) and the within-subject variable congruency (congruent vs. incongruent). See Table 1 for descriptive statistics.

#### Accuracy rates

The expected main effect of congruency was significant, indicating that participants responded more accurately on congruent than incongruent trials, *F*(1, 124) = 364.42, *p* < .001, $\eta_{p}^{2}$ = .75. The highly significant main effect of age group indicated that children committed more errors than adults, *F*(1, 124) = 32.90, *p* < .001, $\eta_{p}^{2}$ = .21. The interaction between congruency and age group was significant, *F*(1, 124) = 22.07, *p* < .001, $\eta_{p}^{2}$ = .15, suggesting a stronger congruency effect for children than for adults. Paired t-tests indicated that the difference between congruent and incongruent trials was significant for children, *t*(62) = 14.902, *p* < .001, as well as for adults, *t*(62) = 11.943, *p* < .001. However, a significant Welch two sample t-test indicated that the difference was indeed larger for children (*M* = .190, *SE* = .013) than for adults (*M* = .115, *SE* = .010), *t*(115.34) = 4.698, *p* < .001.

#### Reaction times

The expected main effect of congruency was significant, indicating that participants responded faster on congruent than incongruent trials, *F*(1, 124) = 420.60, *p* < .001, $\eta_{p}^{2}$ = .77. The highly significant main effect of age group indicated that children responded slower than adults, *F*(1, 124) = 152.24, *p* < .001, $\eta_{p}^{2}$ = .55. The interaction between congruency and age was significant, *F*(1, 124) = 19.65, *p* < .001, $\eta_{p}^{2}$ = .14, suggesting a stronger congruency effect for children than for adults. Paired t-tests indicated that the difference between congruent and incongruent trials was significant for children, *t*(62) = 14.685, *p* < .001, as well as for adults, *t*(62) = 15.221, *p* < .001. However, a significant Welch two sample t-test indicated that the difference was indeed larger for children (*M* = 135, *SE* = 9) than for adults (*M* = 87, *SE* = 6), *t*(103.71) = 4.433, *p* < .001.

### Pure post-error slowing

As shown in Table 2, pure post-error slowing was computed by subtracting reaction times on trials after correct responses from reaction times on trials after incorrect responses on incongruent trials. T-tests indicated that the difference scores were significantly different from 0 on every trial, confirming a post-error slowing effect for both age groups. An ANOVA with age group (children vs. adults) as between subjects variable and trial (t+1, t+2, t+3, t+4) as a within-subjects variable revealed no main effect of age group, *F*(1, 124) = 0.84, *p* = .361, $\eta_{p}^{2}$ < .01, and no interaction with trial, *F*(1.64, 202.87) = 0.44, *p* = .603, $\eta_{p}^{2}$ < .01. The main effect of trial, however, was highly significant, *F*(1.64, 202.87) = 48.07, *p* < .001, $\eta_{p}^{2}$ = .28, suggesting a decline of post-error slowing over the course of the four trials. Bonferroni adjusted post-hoc t-tests revealed that the differences between the first trial and all other trials were significant, all *p*’s < .001. The difference between the second and third trial was also significant, *p* = .05, while the other comparisons were not significant, *p* > .26.

Because lower error rates are sometimes associated with more pronounced post-error slowing [47], a possibly stronger post-error slowing effect in children may have been disguised by the fact that children had higher error rates, thus reducing post-error slowing. To account for different error rates in the age groups, we conducted an ANCOVA including the covariate “accuracy on incongruent trials”. The main effect of age group was indeed significant, *F*(1, 123) = 4.46, *p* = .037, $\eta_{p}^{2}$ = .04, suggesting stronger slowing in children. The main effect of trial was still significant, *F*(1.69, 207.93) = 5.42, *p* = .008, $\eta_{p}^{2}$ = .28, suggesting a decrease in post-error slowing with time. The interaction between age group and trial was also significant, *F*(1.69, 207.93) = 3.59, *p* = .036, $\eta_{p}^{2}$ = .03. The main effect of accuracy rate was also significant, *F*(1, 123) = 8.34, *p* = .005, $\eta_{p}^{2}$ = .06, as well as the interaction with trial, *F*(1.69, 207.93) = 10.36, *p* < .001, $\eta_{p}^{2}$ = .08, suggesting that the accuracy rate affects not only the strength of the effect but also the longevity. However, because the accuracy rate variable is not independent from our main variable age group, the interpretations drawn by this ANCOVA need to be viewed with caution.

### Post-conflict slowing versus post-error slowing

**Fig 3:**
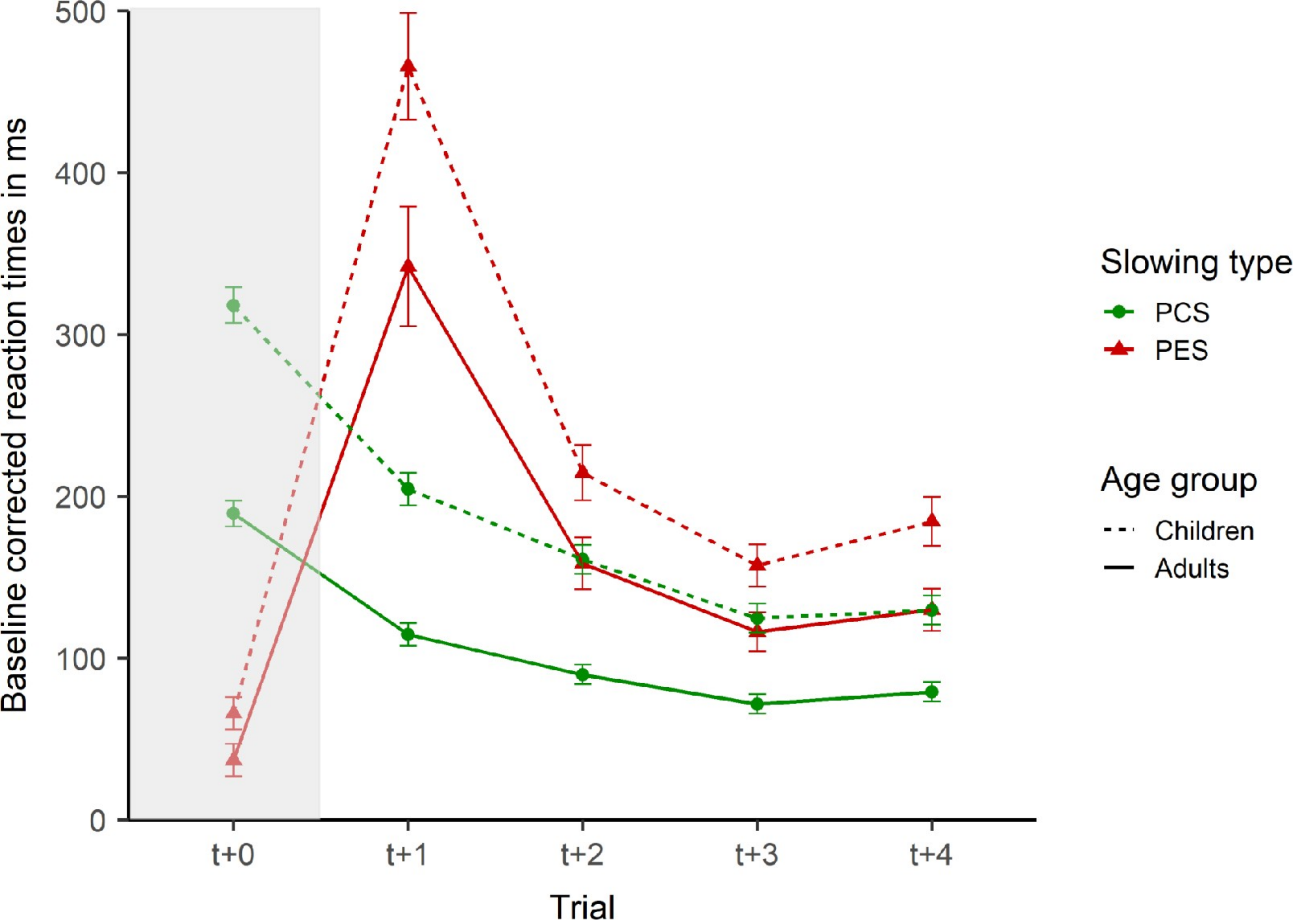
Post-Conflict slowing and post-error slowing. Baseline corrected reaction times of children (dotted line) and adults (solid line) on incongruent trials (t+0) and the following correct congruent trials (t+1, t+2, t+3, t+4). The slowing type depends on the correctness of trial t+0. Post-Conflict Slowing (PCS; green line, circles) follows a correct response and Post-Error Slowing (PES; red lines, triangles) follows an incorrect response. Error bars represent standard errors of the mean.

We calculated post-conflict and post-error slowing by subtracting the individual baseline reaction times (pure congruent block) from the reaction times on four congruent trials after correct and incorrect incongruent trials in the mixed block (see Fig 3 for the resulting difference scores and the trajectory over the four trials). The deviation from zero thus represents the amount of slowing due to the inducement of cognitive conflict and errors. We tested for every trial and separately for adults and children whether slowing was significantly greater than zero. The t-tests indicated significant slowing on every trial, all *p*’s < .001. To examine post-conflict and post-error slowing, we conducted our main ANOVA with age group (children vs. adults) as a between-subjects variable, and slowing type (post-conflict vs. post-error) and trial (t+1, t+2, t+3, t+4) as within-subjects variables. Fig 3 depicts the time courses of post-conflict and post-error slowing. Please note that the incongruent trial (t+0) is presented in Fig 3 for completeness reasons and was not included in the analysis.

Children performed slower than adults (*M* = 205, *SE* = 7 vs. *M* = 138, *SE* = 7) as indicated by a significant main effect of age group, *F*(1, 124) = 27.68, *p* < .001, $\eta_{p}^{2}$ = .18. Both age groups performed slower after errors (*M* = 221, *SE* = 9 vs. *M* = 122, *SE* = 3), as indicated by a significant main effect of slowing type, *F*(1, 124) = 174.28, *p* < .001, $\eta_{p}^{2}$ = .58. Reaction times decreased from the first (*M* = 282, *SE* = 15) to the second (*M* = 156, *SE* = 7), and to the third (*M* = 117, *SE* = 5) post trial, with reaction times increasing towards the fourth trial (*M* = 131, *SE* = 6), as indicated by a significant main effect of trial, *F*(1.62, 201.21) = 103.47, *p* < .001, $\eta_{p}^{2}$ = .45. Pairwise comparisons with the Bonferroni correction for multiple comparisons indicated significant decreases in slowing from t+1 to t+3, all *p*’s < .001, but an insignificant increase from t+3 to t+4, *p* = .144.

The interaction between age group and slowing type was not significant, *F*(1, 124) = 0.03, *p* = .858, $\eta_{p}^{2}$ < .01, suggesting comparable reaction time adjustments after conflicts and errors for both age groups. However, the interaction between age group and trial was significant, *F*(1.62, 201.21) = 3.36, *p* = .047, $\eta_{p}^{2}$ = .03, suggesting age related differences in the time course of reaction time adjustments. Although children had stronger slowing on every trial, the difference was largest on t+1 (*M* = 107, *SE* = 27) compared to the other trials t+2 (*M* = 64, *SE* = 15), t+3 (*M* = 47, *SE* = 12), and t+4 (*M* = 53, *SE* = 14). Furthermore, the interaction between slowing type and trial was highly significant, *F*(1.57, 194.63) = 49.54, *p* < .001, $\eta_{p}^{2}$ = .29, suggesting a different time course for reaction time adjustments after conflicts and errors. Although post-error slowing was stronger than post-conflict slowing on every trial, all *p*’s < .001, the difference on the first trial was significantly largest compared to all other trials, all *p*’s < .001. The other trial comparisons of the difference between post-conflict and post-error slowing were not significant, all *p*’s > .053. The three-way interaction was not significant, *F*(1.57, 194.63) = 0.65, *p* = .486, $\eta_{p}^{2}$ < .01.

## Discussion

The present study compared the time course of adjustments following cognitive conflict and errors. Preadolescent children and young adults performed a Simon task, in which every fifth trial was incongruent. These incongruent trials were designed to experimentally provoke conflict and errors. Reaction times were examined on the subsequent four congruent trials as a function of whether the response on the previous incongruent trial was correct (post-conflict) or incorrect (post-error). Compared to a baseline block consisting of only congruent trials, both age groups showed substantial slowing for several trials after conflicts and errors. This long-lasting slowing effects are in line with previous studies investigating post-conflict slowing or post-error slowing [2,5,8,9]. For the first time, the two slowing phenomena were compared directly by using the same analytical approach. Results indicated that both age groups showed stronger post-error than post-conflict slowing. The difference in slowing represents the pure post-error slowing effect and suggests that participants noticed the error and adjusted performance [48,49]. The additional slowing after an error was most pronounced on the first trial. According to the conflict monitoring theory, this finding suggests that participants experienced an additional response conflict between the correct and incorrect response [17]. Apart from this delayed conflict detection, the time-course was comparable to post-conflict slowing. As the age effect (i.e., stronger slowing in children) was also comparable between the two slowing phenomena, we suggest that post-conflict slowing and post-error slowing share underlying processes.

Overall, children slowed down more strongly than adults. Due to the baseline correction, this slowing does not solely reflect slower information processing [50]. It rather reflects more monitoring costs and/or coarser performance adjustments after conflicts and errors [8,9]. More slowing has been shown to emerge in conditions of higher control demands [51,52]. As the higher error rates and stronger congruency effects indicate, the mixed block was indeed more demanding for the children than for the adults. The relative higher cognitive load may have led to more monitoring for the expected conflict trials and thus more slowing. Assuming an involvement of an affective component in conflict and error monitoring, the higher cognitive load may elicit a stronger affective response in children leading to stronger slowing [53,54]. On the other hand, less developed performance adjustment skills would lead to stronger after-effects of responding (correctly or incorrectly) to a conflict trial [55]. Thus, we suspect that more monitoring costs as well as less developed performance adjustment skills contributed to the performance differences between children and adults. With better adjustment skills there is less need to engage in costly monitoring.

The larger post-conflict slowing effect in children is consistent with findings showing reduced interference with age and is in line with the theory of increasing ability to inhibit interference and to self-regulate [45,46,56]. A recent study suggests that congruent trials affect sequential control adaptation by relaxing cognitive control [57]. It may be that the stronger post-conflict slowing in children is not only due to slower performance adaptations after conflicting incongruent trials, but it may also be that children are slower in relaxing cognitive control after encountering congruent trials. We investigated post-conflict slowing on four subsequent congruent trials and on all four trials post-conflict slowing was stronger in children than in adults. It is an open debate whether this longevity of the age effect is due to stronger and longer lasting disturbance from conflicts or whether it is due to children’s slower relaxation of cognitive control in the face of congruent trials.

The quasi-experimental nature of the research on post-error slowing raised debates on methodological, empirical and theoretical grounds. For example, it has been found that the inter-trial interval affects the magnitude of post-error slowing, with shorter intervals typically leading to larger slowing effects [8,16,25,58–60]. Some studies report reduced post-error slowing with age, in line with the theory of improving cognitive control [9,10,31,32,41]. However, the findings are mixed [8]. Some studies did not find any age related change [33,35,37,61] and others found even an age-correlated increase in post-error slowing [36]. Differences in error rates between age groups may obscure true age effects. As highly accurate individuals exhibit largest post-error slowing [47] and adults are typically more accurate than children, adult’s post-error slowing is relatively increased compared to children’s. Furthermore, the traditional measure of post-error slowing compares mean reaction times for post-correct and post-error trials. This aggregated measure may not be sensitive to differences in fine-grained performance adjustments across trials [9,40]. Our results support this interpretation by showing the expected decrease in slowing with age using the method inspired by research on post-conflict slowing while the traditional measure of post-error slowing yielded no significant age differences. However, when controlling for error rates by means of a covariance analysis we found the expected age effect even with this method.

Accounts of strategic monitoring and adjustment of performance assume increased response caution and increased cognitive control after errors and predict that post-error slowing is accompanied by a reduction of interference and accuracy increase. This is exactly what some studies using relatively long inter-trial intervals found [34,62–64]. However, other studies using a relatively short inter-trial interval found a post-error accuracy decrease [65,66]. These studies support the orienting account of post-error slowing which assumes that an automatic orienting response to the error and away from the current task is responsible for decreased post-error performance [67]. Studies varying the inter-trial interval found that timing is critical for the emergence and direction of post-error changes in performance [25,58]. Thus, the two accounts are not mutually exclusive [16]. Rather, their combination may explain more empirical findings.

The present results are best explained with two-process theories. According to Wessel’s *adaptive orienting theory of error processing* errors first invoke processes inhibiting ongoing behavior and orienting attention toward the source of the error after which more controlled error-specific processes adjust the existing task set [68]. As response inhibition is not fully matured in preadolescent children this may explain the stronger slowing in children compared to adults [45]. Thus, our results support inhibition accounts of post-error slowing [69,70]. While responses are inhibited the error captures attention because it is an unexpected event and thus this account may also apply to occasional incongruent trials [67]. Orienting attention to the error leads to a co-activation of the given incorrect response and the required correct response. We propose that errors induce response conflict on the first subsequent trial. This assumption is supported by the result of very fast reaction times on error trials and very slow reaction times immediately after the error. In other words, errors are followed by a cognitive conflict triggered by the realization of the incompatibility of the given incorrect response and the required correct response. The incompatibility of the two responses induces response conflict similar to the response conflict induced by incongruent trials [17,22].

The advantage of the presented method is the possibility to compare post-conflict slowing and post-error slowing with the same analytical approach. However, there are also drawbacks to this method. One limitation is the relatively low trial number. In experimental cognitive research a lot of trials are used to increase power. Especially in research on post-error slowing a lot of trials are needed to ensure enough post-error trials. However, in developmental research lengthy tasks are not suitable as children’s motivation and attention declines over a longer period of repetitive trials. Thus, we aimed to come up with a task that was short enough to engage and keep children focused on the task while at the same time ensuring that the task elicits enough errors in adults. We opted for a shorter task and compensated for the loss in power with a relatively large sample size [71]. However, the problem of differing error rates in children and adults is not solved. As the error rate is found to influence post-error slowing [47], future studies could match children and adults on error rates. Another limitation related to the shortness of the task is the fixed block order. By presenting the pure congruent block first, we aimed at increasing the congruency effect and thus increasing the error rate on incongruent trials. Including more blocks and balancing block order is another direction for future studies.

Taken together, occasional incongruent trials in the Simon task elicit cognitive conflicts leading to slower and more error-prone responses on this incongruent trials. Performance on the subsequent congruent trials is slowed and even more so if an error happened. We propose the same processes underlying post-conflict and post-error slowing just displaced in time. A first inhibitory process hinders fast and inappropriate responses and a second process regulates the speed-accuracy trade-off by an upregulation of cognitive control [9,17,28]. Successful response inhibition leads to a correct response after which the consequences of the speed-accuracy regulation are still observable as longer reaction times (post-conflict slowing). If, however, response inhibition fails on an incongruent trial, it is likely that an error occurs. This error is characterized by fast reaction times and entails a delayed, yet strong response inhibition leading to post-error slowing [69,70]. Both slowing phenomena last for several trials reflecting the upregulation of cognitive control [17].

In conclusion, we propose that the same processes underlie post-error slowing and post-conflict slowing but they are displaced in time (cf. Fig 3). The effect of the first process dissipates quickly while the second process has longer lasting effects. Previous research on conflict-slowing and post-error slowing suggest that the first process is age invariant but time sensitive, while the second process is sensitive to age but not to time [8,25–29]. Further studies are needed to explain in detail how conflict and error processing are supposed to differ functionally and anatomically if the timing of the involved processes is taken into account [4,23,24,72,73]. The presented method allows differentiating between different processes experimentally and investigating cognitive development in more detail.
